# Bioaccumulation and Haematological Alterations Induced by Varying Concentrations of Cadmium in Selected Organs of Golden Misri Chickens (*Gallus gallus domesticus*) in Khyber Pakhtunkhwa, Pakistan

**DOI:** 10.1021/acsomega.5c03393

**Published:** 2025-08-04

**Authors:** Uzma Yousaf, Ali Muhammad Yousafzai, Waseem Danish, Muhammad Umar, Alevcan Kaplan, Esra Gökçe Gündüzer, Yasir Assad

**Affiliations:** † Department of Zoology, 124261 Islamia College Peshawar, Peshawar 25120, Khyber Pakhtunkhwa, Pakistan; ‡ Department of Animal Nutrition, 66936 The University of Agriculture Peshawar, 25130 Peshawar, Pakistan; § Department of Animal Sciences, 66757 Quaid-i-Azam University, Islamabad 45320, Pakistan; ∥ Department of Crop and Animal Production, Sason Vocational School, 187432 Batman University, Batman 72060, Turkey; ⊥ Department of Biology, Faculty of Science, 37511 Gazi University, Ankara 06500, Turkiye; # Department of Zoology, 66934 Hazara University, Mansehra 21300, Pakistan

## Abstract

The aim of the research was to evaluate the effects of Cadmium (Cd) toxicity on hematological parameters and its bioaccumulation in Golden Misri chickens (*Gallus gallus* domesticus), focusing on both dosage and exposure duration. The study was conducted from June to September 2021 at the Zoology Department of Islamia College Peshawar and included 60 sexually mature Golden Misri chickens, each weighing approximately 1 kg and aged 8 weeks. The chickens were systematically divided into two batches for short-term (3 and 5 days) and long-term exposure studies (14, 20, 40, and 60 days). Sublethal oral doses of Cd were administered to the experimental groups: 35 mg/kg and 30 mg/kg for 3- and 5 day trials, respectively, and 25 mg/kg, 20 mg/kg, 15 mg/kg, and 10 mg/kg for long-term trials of 14, 20, 40, and 60 days. The blood and tissue samples were analyzed using an atomic absorption spectrophotometer and a hematological analyzer, with statistical significance at *p* < 0.05. The results demonstrated that while short-term exposure caused initial Cd accumulation and mild hematological changes, long-term exposure led to a progressive and significantly higher accumulation of Cd in the liver and kidneys, with severe hematological alterations. Hematological analysis showed a significant reduction in red blood cell (RBCs) (erythrocytes), hemoglobin (Hb), Hematocrit (HCT), mean corpuscular volume (MCV), mean corpuscular hemoglobin (MCH), mean corpuscular hemoglobin concentration (MCHC), and platelets (PLT), accompanied by an increase in white blood cell (WBCs) compared to the control group. These findings confirm a duration-dependent pattern of Cd toxicity, with increased exposure time contributing to higher tissue accumulation and greater systemic effects. The study concludes that prolonged Cd exposure significantly alters key hematological markers, contributing to systemic toxicity in Golden Misri chickens.

## Graphical abstract

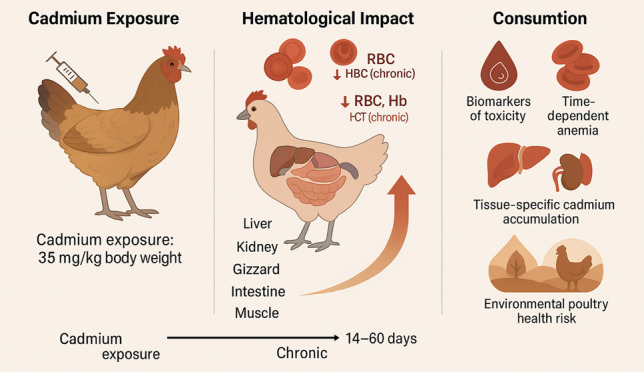

## 1 Introduction

In recent decades, chickens have been increasingly exposed to alarming levels of heavy metals, particularly through their feed and water, a trend that has been exacerbated by increasing human industrial activities.^1^ Among these contaminants, Cd is a substance of high concern due to its high toxicity and persistence in biological systems. The United States (US) Agency for Toxic Substances and Disease Registry categorizes Cd as one of the most hazardous substances,^2,3^ Its exceptionally long biological half-life of 10 to 30 years and its limited excretion make it particularly harmful to bird species such as chickens.^4^ Cd accumulates primarily in the liver and kidneys, and poses a significant health hazard not only to poultry but also to humans who consume it.^5^ The toxicological profile of Cd is exacerbated by its propensity to bioaccumulate and biomagnify in the food chain, making threat to public health.^6^ Prolonged exposure and excessive accumulation of Cd can lead to severe physiological disorders, including hepatic and renal dysfunction and reproductive impairment.^7^ Empirical evidence from experimental studies has consistently demonstrated that feed or water contaminated with Cd leads to a significant accumulation of this heavy metal in the liver and kidneys of avian species such as ducks, chickens, and Japanese quail, emphasizing the serious ecological and health consequences of Cd exposure.^8^

Chronic exposure to Cd triggers a cascade of pathological alterations in chickens, manifested by significant organ accumulation and disturbances of hematobiochemical parameters. Cd is a pervasive environmental pollutant that enters ecosystems from both natural and industrial sources; however, anthropogenic activities, particularly industrialization and agricultural practices, contribute three to ten times more to Cd exposure than natural processes.^9^ When, Cd concentrations exceed the binding affinity of metallothionein, an essential metal-binding protein, unbound Cd triggers oxidative stress through the formation of lipid peroxides and free radicals. This oxidative damage primarily affects critical organs, such as the kidneys and liver, leading to severe functional impairment.^10^ Increased Cd intake in poultry is associated with a myriad of negative effects, including a significant decrease in egg production, reduced feed intake, and increased susceptibility to environmental and physiological stressors.^11^ The toxic metal interferes with essential metabolic processes, particularly affecting protein concentrations, which are important for digestion and nutrient transport. This in turn impairs the excretory function in the oviduct of the chicken, leading to further systemic dysfunction.^12^ Cd ions tend to bind to high-molecular-weight proteins, which are mainly found in RBC. However, Hb contains only a small proportion of circulating the, indicating a complex interplay between Cd distribution of Cd and its toxic dynamics in avian physiology. Consequently, the bioaccumulation of Cd, together with its ability to induce oxidative stress and impair organ function, emphasizes its profound threat to both avian health and broader ecological systems.

Hematological parameters such as (RBCs, erythrocytes), (WBCs, leukocytes), Hb, (HCT), (PLT), (MCV), (MCH), and (MCHC) serve as fundamental biomarkers for assessing the health, function, and integrity of blood. The World Health Organization (WHO) underscores the usefulness of these hematological parameters as critical tools for both medical diagnosis and nutritional assessment, providing insights into an individual’s systemic health.^13^ These indicators are highly sensitive to a variety of internal and external factors, including physiological state, food intake, dietary and, water restrictions, fasting, age-related variabilities, and notably, exposure to heavy metals such as Cd.^14^ Cd exposure has been shown to cause profound alterations in these blood parameters, contributing to significant hematobiochemical disturbances that go beyond a simple metabolic disorder and have potentially detrimental effects on overall physiological well-being.^15^ The hematopoietic system, which is responsible for the production of blood cells, is acutely sensitive to Cd toxicity, making the blood not only a medium for Cd transport but also a major target for its toxic effects.^16^ Impairment of erythropoiesis, the process of RBC formation, by Cd can lead to anemia via several pathogenic mechanisms, including inhibition of erythrocyte production, hemolysis, and suppression of essential hematopoietic factors.^17^ Furthermore, the ability of Cd to induce cell necrosis and apoptosis in critical tissues underscores its designation as a Group 1 carcinogen by the International Agency for Research on Cancer (IARC), due to its established potential to trigger carcinogenesis through genomic instability, oxidative damage, and promotion of malignant cell proliferation.^18^ The bio accumulative nature of Cd, its disruption of vital hematological parameters, and its ability to cause both acute and chronic health issues solidify its status as a serious threat to the environment and public health. The impacts of Cd exposure on the blood system, combined with its broader systemic effects, make it an insidious toxin with far-reaching consequences for individual and ecological health.

Golden Misri chickens were selected for this study due to their widespread use in South Asian poultry production, high adaptability, and economic value. Their exposure to commercial feed and water makes them a suitable model for assessing environmental toxin effects. Cadmium (Cd), a known bioaccumulative and Group 1 carcinogen (IARC), was chosen due to its prevalence in agricultural and industrial pollutants. The study included both acute (3 and 5 days) and chronic (14, 20, 40, and 60 days) exposure durations to distinguish between immediate and progressive toxic effects. Sublethal doses ranging from 35 to 10 mg/kg were selected based on prior research to assess dose–response relationships. The objective was to evaluate tissue-specific Cd accumulation and its impact on hematological parameters across different exposure periods.

## 2 Materials and Methods

### 2.1 Ethical Statement

All procedures involving animals were conducted in accordance with ethical standards and approved by the Graduate Study Committee of Islamia College Peshawar, Pakistan (Ethics Approval no: ICP/GSC/Zoo/127-2021). The study strictly adhered strictly to institutional guidelines to ensure the humane treatment and welfare of all experimental animals.

### 2.2 Chickens and Chemical Collection and Management

The present study was meticulously conducted from June to September 2021 in the animal facility of the zoology department of, Islamia College Peshawar. Golden Misri chickens (*Gallus gallus domesticus*) were carefully selected as subjects. All chickens were 8 weeks old at the beginning of the study and had an average weight of approximately 1 kg.

The chickens were fed a standard poultry diet sourced from a certified local feed supplier (Jadeed Feeds Pvt. Ltd., Pakistan). The feed contained 18% crude protein, 2800 kcal/kg metabolizable energy, 3% crude fat, 5% crude fiber, 1% calcium, and 0.45% available phosphorus. The diet was fed three times a day (morning, noon, and evening) and the animals had ad libitum access to clean water. A cohort of 60 mature, robust, and healthy chickens, uniform in age and weight, and with no visible signs of illness, was procured from the local market in Peshawar. The 60 chickens were randomly divided into six treatment groups and one control group, each consisting of 10 chickens (*n* = 10 per group per time point). The treatment groups received Cd doses of 35, 30, 25, 20, 15, and 10 mg/kg for 3, 5, 14, 20, 40, and 60 days respectively, while the control group was not exposed to Cd. To facilitate the acclimatization of the experimental chickens and ensure their welfare, they were housed in enclosures designed to provide natural light and thus allow both acoustic and visual interactions between the birds. The chickens were kept under standard nutritional conditions, had ad libitum access to water and were adequately ventilated. Their health was continuously monitored through vigilant observation of physical behavior and activity levels. The Golden Misri chickens were fed a cereal mixture of wheat, crushed rice, and other grains, three times a day (morning, noon, and evening). The cages were regularly cleaned and disinfected to maintain strict hygiene standards. Throughout the experimental period, clinical signs, stress indicators, and behavioral changes were rigorously evaluated in all groups, with assessments being carried out twice daily and systematically classified into mild, moderate, and severe categories. Additionally, mean body weights and food intake were meticulously recorded to facilitate a comprehensive comparison between the treatment groups and the control group. Mortality rates were closely monitored, and chickens were fasted for approximately 12 h prior to blood sampling to ensure uniform hematological analysis.

Analytical grade Cadmium chloride (CdCl_2_) was used in this study. The chemical was purchased from Sigma-Aldrich (Merck KGaA, Darmstadt, Germany), under catalogue number 202908 and batch number MKCK8042. All solutions were prepared with distilled water, and glassware was thoroughly acid-washed and rinsed to avoid contamination.

### 2.3 Preparation of Cadmium Chloride Stock Solution

A cadmium chloride solution was used as a test solution in the experiments. To prepare a stock solution with a Cd concentration of 1000 mg/L (1000 ppm) of Cd, 1.63 g of CdCl_2_ was dissolved in 1 L of distilled water, as this amount of CdCl_2_ contains approximately 1 g of Cd. After preparation, the stock solution should be stored in a clean glass bottle to maintain its stability and effectiveness. This method ensures the preparation of an accurate 1000 mg/L Cd solution.

### 2.4 Experimental Design

The experiment was conducted in two batches, comprising both short-term and long-term exposure trials. The short-term trials spanned 3 and 5 days, while the long-term trials lasted 14, 20, 40, and 60 days. Oral sub lethal doses of Cd were administered to the test groups, namely 35 mg/kg and 30 mg/kg for the 3 day- and 5 day trials, respectively. In the long term trials, Cd was administered at doses of 25 mg/kg, 20 mg/kg, 15 mg/kg, and 10 mg/kg over a period of 14, 20, 40, and 60 days, respectively. The experimental setup followed a completely randomized design (CRD), in which all chickens were randomly assigned to a treatment and a control group without blocking. The Cd doses (35, 30, 25, 20, 15, and 10 mg/kg body weight) were selected based on previous toxicological studies in poultry and avian models in which similar concentrations produced sub lethal physiological and hematological effects without inducing mortality.^11,19^ These references guided the choice of a descending dose range to allow evaluation of both acute and chronic exposure responses. No separate LD_50_ or dose–response study was conducted for this experiment.

### 2.5 Tissue Digestion and Estimation of Cadmium

Frozen tissue samples of intestine, liver, muscle, kidney, and gizzard were thawed, washed with distilled water, and then desiccated with blotting paper. The measured weights of these tissues from each chicken were transferred to 250 mL volumetric flasks for the digestion process. Digestion was performed according to the methods of, Van Loon, (2012)^20^ and Du Preez, and Steyn (1992)^21^ with a slight variation: instead of the initial addition of 10 mL of 55% nitric acid and 5 mL of 70% perchloric acid, 5 mL of 55% nitric acid and 1 mL of 70% perchloric acid were added to each flask and allowed to stand overnight. The next day, a further 5 mL of 55% nitric acid and 4 mL of 70% perchloric acid were added. The flasks were then placed on a hot plate and heated to 200 and 250 °C until a transparent solution was formed. The appearance of thick white vapors following brown fumes indicated that digestion was complete. This method reduced the digestion time to about 20 min, compared to the 3 to 4 h reported by Van Loon, (2012).^20^ Following digestion, the residues were allowed to cool, diluted to 10 mL with distilled water, and the flasks were rinsed thoroughly.

### 2.6 Collection and Preservation of Blood

Blood samples were collected from the pectoral vein of each chicken using sterile syringes and placed in pre labeled EDTA-coated tubes to prevent coagulation. The EDTA concentration used was 2 mg/mL blood. The tubes were gently inverted several times to ensure proper mixing without hemolysis. The samples were immediately stored in an ice box and transported to the laboratory for hematological analysis. Until analysis, all samples were stored in a refrigerator at 4 °C to maintain cellular integrity.

### 2.7 Hematological Analysis

Hematological parameters were analyzed using a Sysmex KX-21 automated hematology analyzer (Sysmex Corporation, USA). The following parameters were measured: WBC*s*, (RBC*s*), Hb concentration, HCT, MCV, MCH, MCHC, and platelet count (PLT). All analyses were performed according to the manufacturer’s protocols and standard laboratory procedures to ensure the accuracy and reproducibility of the data.

### 2.8 Statistical Analysis

All experimental data were expressed as mean ± standard error (S.E.). One-way analysis of variance (ANOVA) was used to determine statistically significant differences between multiple treatment groups exposed to different Cd concentrations and durations. The assumptions of normality and homogeneity of variances were tested using the Shapiro–Wilk test and Levene test, respectively. A *p*-value of <0.05 was considered statistically significant. All analyses were performed using IBM SPSS Statistics software (version 25.0, IBM Corp., Armonk, NY, USA).

## 3 Results

In the present study, the effects of Cd toxicity on hematological parameters in the blood and the accumulation of Cd in selected tissues were evaluated on the basis of dose and duration in the Golden Misri chicken.

### 3.1 Cadmium Bioaccumulation in Various Tissues

The accumulation of Cd in various tissues of *G*. *gallus domesticus* after 3 days of exposure to 35 mg/kg Cd is shown in Table 1. Significant accumulation (*p* < 0.05) was observed in the kidney (0.051 ± 0.0006), liver (0.038 ± 0.0009), gizzard (0.021 ± 0.00115), and muscle (0.015 ± 0.00057). The intestine showed nonsignificant accumulation (0.024 ± 0.001, *p* = 1.000). The accumulation order was kidney > liver > intestine > gizzard > muscles. After a 5 day exposure to 30 mg/kg Cd, there was significant accumulation (*p* < 0.05) was observed in the liver (0.131 ± 0.00088), kidney (0.036 ± 0.00115), and gizzard (0.031 ± 0.0012), as shown in Table 2. Nonsignificant accumulation was found in the intestine (0.027 ± 0.0012, *p* = 0.140) and muscles (0.005 ± 0.0017, *p* = 0.440). The order of Cd accumulation was liver > kidney > gizzard > intestine > muscle. During a 14 day exposure to 25 mg/kg Cd, significant Cd accumulation (*p* < 0.001) was observed in the liver (0.163 ± 0.0012) and kidney (0.078 ± 0.0008), as shown in Table 3. Nonsignificant accumulation was found in the gizzard (0.075 ± 0.00115, *p* = 0.090), intestine (0.058 ± 0.0009, p = 0.060), and muscles (0.020 ± 0.00577, *p* = 0.131). The accumulation sequence was liver > kidney > gizzard > intestine > muscle. In a 20 day study with 20 mg/kg Cd exposure, significant Cd accumulation (*p* < 0.05) was observed in the liver (0.127 ± 0.00088) and kidney (0.124 ± 0.00115), as shown in Table 4. Nonsignificant accumulation was found in the intestine (0.055 ± 0.00057, *p* = 0.076), gizzard (0.038 ± 0.0012, *p* = 0.060), and muscles (0.006 ± 0.0012, *p* = 0.609). The accumulation sequence was liver > kidney > intestine > gizzard > muscle. After a 40 day exposure to 15 mg/kg Cd, significant accumulation was observed in the kidney (0.215 ± 0.00057, *p* < 0.001), liver (0.204 ± 0.00115, *p* < 0.05), and muscles (0.013 ± 0.0012, *p* = 0.027), as shown in Table 5. Nonsignificant accumulation was found in the intestine (0.091 ± 0.0008, *p* = 0.090) and gizzard (0.042 ± 0.001, *p* = 0.212). The accumulation sequence was kidney > liver > intestine > gizzard > muscle. Following 60 days of exposure to 10 mg/kg Cd, there was a significant Cd accumulation (*p* < 0.001) was observed in the kidney (0.311 ± 0.00088) and liver (0.243 ± 0.012), as shown in Table 6. Nonsignificant accumulation was noted in the gizzard (0.134 ± 0.00057, *p* = 0.060), intestine (0.122 ± 0.00057, *p* = 0.080), and muscles (0.012 ± 0.0012, *p* = 0.185). The sequence of Cd accumulation was kidney > liver > gizzard > intestine > muscle.

**Table 1:** Showing Cd Bioaccumulation in Selected Tissues of Both Control and Treated *G. gallus domesticus* Following 3 Day Oral Intoxication with 35 mg/kg Body Weight Compared to the Control Group^a^

tissues	control	treated	*P* value
liver	0.032 ± 0.0009	0.038 ± 0.0009	0.039
kidney	0.032 ± 0.00115	0.051 ± 0.0006	0.042
gizzard	0.015 ± 0.0008	0.021 ± 0.00115	0.018
intestine	0.024 ± 0.0006	0.024 ± 0.001	1.000
muscles	0.007 ± 0.00115	0.015 ± 0.0057	0.030

a All the values are expressed as mean ± S.E, based on five chickens per group. Statistical significance was determined using Student’s *t*-test: (*p* < 0.05) significant, (*p* < 0.01) more significant, and (*p* < 0.001) highly significant, compared to the control group.

**Table 2:** Bioaccumulation of Cd in *G. gallus domesticus* Following 5 Day Oral Intoxication with 30 mg/kg Body Weight Compared to Control Group^a^

tissues	control	treated	*P* value
liver	0.053 ± 0.00881	0.131 ± 0.00088	0.011
kidney	0.018 ± 0.00120	0.036 ± 0.00115	0.020
gizzard	0.011 ± 0.0010	0.031 ± 0.0012	0.023
intestine	0.024 ± 0.0012	0.027 ± 0.0012	0.140
muscles	0.003 ± 0.0008	0.005 ± 0.0017	0.440

a All the values are expressed as mean ± S.E, based on five chickens per group. Statistical significance was determined using Student’s *t*-test: (*p* < 0.05) significant, (*p* < 0.01) more significant, and (*p* < 0.001) highly significant, compared with the control group.

**Table 3:** Cd Bioaccumulation in *Gallus domesticus* Following 14 Day Oral Intoxication with 25 mg/kg Body Weight Compared to the Control Group^a^

tissues	control	treated	*P* value
liver	0.075 ± 0.00088	0.163 ± 0.0012	0.000
kidney	0.026 ± 0.00115	0.078 ± 0.0008	0.000
gizzard	0.034 ± 0.00057	0.075 ± 0.00115	0.090
intestine	0.046 ± 0.0006	0.058 ± 0.0009	0.060
muscles	0.009 ± 0.00057	0.020 ± 0.00577	0.131

a All values are expressed as mean ± SE based on five chickens per group. Statistical significance was determined using Student’s *t*-test: (*p* < 0.05) significant, (*p* < 0.01) more significant, and (*p* < 0.001) highly significant, compared to the control group.

**Table 4:** Cd Bioaccumulation in *G. gallus domesticus* Following 20 Day Oral Intoxication with 20 mg/kg Body Weight Compared to Control group^a^

tissues	control	treated	*P* value
liver	0.038 ± 0.00176	0.127 ± 0.00088	0.045
kidney	0.039 ± 0.00057	0.124 ± 0.00115	0.030
gizzard	0.034 ± 0.00115	0.038 ± 0.0012	0.060
intestine	0.024 ± 0.00057	0.055 ± 0.00057	0.076
muscles	0.005 ± 0.0020	0.006 ± 0.0012	0.609

a All values are expressed as mean ± S.E, based on five chickens per group. Statistical significance was determined using Student’s *t*-test: (*p* < 0.05) significant, (*p* < 0.01) more significant, and (*p* < 0.001) highly significant, compared to the control group.

**Table 5:** Cd Bioaccumulation in *G. gallus domesticus* Following 40 Day Oral Intoxication with 15 mg/kg Body Weight Compared to Control Group^a^

tissues	control	treated	*P* value
liver	0.046 ± 0.00088	0.204 ± 0.00115	0.045
kidney	0.056 ± 0.00057	0.215 ± 0.00057	0.000
gizzard	0.031 ± 0.011	0.042 ± 0.001	0.212
intestine	0.043 ± 0.00057	0.091 ± 0.0008	0.090
muscles	0.008 ± 0.001	0.013 ± 0.0012	0.027

a All values are expressed as Mean ± S.E, based on five chickens per group. Statistical significance was determined using Student’s *t*-test: (*p* < 0.05) significant, (*p* < 0.01) more significant, and (*p* < 0.001) highly significant, compared to the control group.

**Table 6:** Cd Bioaccumulation in *G. gallus domesticus* Following 60 Day Oral Intoxication with 10 mg/kg Body Weight Compared to Control Group^a^

tissues	control	treated	*P* value
liver	0.067 ± 0.001	0.243 ± 0.012	0.050
kidney	0.063 ± 0.00115	0.311 ± 0.00088	0.000
gizzard	0.042 ± 0.00088	0.134 ± 0.00057	0.060
intestine	0.047 ± 0.0015	0.122 ± 0.00057	0.080
muscles	0.009 ± 0.00115	0.012 ± 0.0012	0.185

a All values are expressed as mean ± S.E, based on five chickens per group. Statistical significance was determined using Student’s *t*-test: (*p* < 0.05) significant, (*p* < 0.01) more significant, and (*p* < 0.001) highly significant, compared to the control group.

### 3.2 Cadmium Effects on Hematological Parameters

The study investigated the Cd toxicological effects of Cd on hematological indicators, and showed varying impacts with time and concentration, as shown in Tables 7–12.

**Table 7:** Hematological Blood Values in *G. gallus domesticus* after 3 Day Oral Exposure to 35 mg/kg Cd Compared to the Control Group^a^

parameters (units)	control	treated	*P* value
RBC × 10^6^/μL	2.73 ± 0.052	2.88 ± 0.0519	0.118
WBC(TLC) × 10^3^/μL	4.26 ± 0.008	4.98 ± 0.0115	0.070
hemoglobin HGB g/dL	11.27 ± 0.012	12.10 ± 0.208	0.016
HCT(PCV) %	27.71 ± 0.540	29.44 ± 0.558	0.090
platelet count × 10^3^/μL	3.84 ± 0.078	3.56 ± 0.220	0.676
MCH pg	36.25 ± 0.565	37.36 ± 1.132	0.427
MCHC pg	33.27 ± 0.589	34.43 ± 1.132	0.323
MCV fL	82.49 ± 0.906	84.23 ± 1.132	0.296

a All the values are expressed as mean ± S.E, based on five chickens per group. Statistical significance was determined using Student’s *t*-test: (*p* < 0.05) significant, (*p* < 0.01) more significant, and (*p* < 0.001) highly significant, compared to the control group.

**8:** Hematological Blood Values in *G. gallus domesticus* after 5 Day Oral Exposure to 30 mg/kg Cd Compared to the Control Group^a^

parameters (units)	control	treated	*P* value
RBC × 10^6^/μL	3.18 ± 0.015	3.74 ± 0.025	0.011
WBC(TLC)×10^3^/μL	3.73 ± 0.135	5.02 ± 0.008	0.001
hemoglobin (Hb) g/dL	12.38 ± 0.351	13.33 ± 0.318	0.116
HCT(PCV) %	29.17 ± 0.580	31.47 ± 0.669	0.060
platelet count × 10^3^/μL	3.51 ± 0.246	3.13 ± 0.227	0.321
MCH pg	35.36 ± 0.577	37.24 ± 0.574	0.082
MCHC pg	32.71 ± 0.606	34.53 ± 0.946	0.181
MCV fL	84.45 ± 0.568	86.63 ± 1.148	0.193

a All values are expressed as mean ± S.E, based on five chickens per group. Statistical significance was determined using Student’s *t*-test: (*p* < 0.05) significant, (*p* < 0.01) more significant, and (*p* < 0.001) highly significant, compared to the control group. Abbreviations, RBC: Red blood cells, (WBC); White blood cell, Hb, HCT/PCV: Hematocrit/packed cell volume, PLT: Platelet count, MCH: Mean corpuscular Hb, MCHC: Mean corpuscular Hb concentration, MCV: Mean corpuscular volume.

**9:** Hematological Blood Values in *G. gallus domesticus* after 14 Day Oral Exposure to 25 mg/kg Cd Compared to the Control Group^a^

parameters (Units)	control	treated	*P* value
RBC × 10^6^/μL	2.93 ± 0.029	2.45 ± 0.020	0.000
WBC(TLC)×10^3^/μL	3.46 ± 0.0145	4.77 ± 0.115	0.042
hemoglobin Hb g/dL	12.37 ± 0.327	11.36 ± 0.279	0.078
HCT(PCV) %	28.83 ± 0.551	24.47 ± 0.263	0.002
platelet count × 10^3^/μL	3.71 ± 0.020	2.88 ± 0.075	0.090
MCH pg	36.23 ± 1.270	34.02 ± 0.560	0.186
MCHC pg	30.35 ± 0.589	28.17 ± 0.438	0.041
MCV fL	80.81 ± 0.605	77.43 ± 0.617	0.017

a All values are expressed as mean ± S.E, based on five chickens per group. Statistical significance was determined using Student’s *t*-test: (*p* < 0.05) significant, (*p* < 0.01) more significant, and (*p* < 0.001) highly significant, compared to the control group. Abbreviations: RBC—Red blood cells (×10^6^/ μL), WBC (TLC)—White blood cells or total leukocyte count (×10^3^/ μL), Hb—Hb (g/dL), HCT (PCV)—Hematocrit or packed cell volume (%), PLT—Platelet count (×10^3^/ μL), MCH—Mean corpuscular Hb (pg), MCHC—Mean corpuscular Hb concentration (pg), MCV—Mean corpuscular volume (fL).

**10:** Hematological Blood Values in *G. gallus domesticus* after 20 Day Oral Exposure to 20 mg/kg Cd Compared to the Control Group^a^

parameters (units)	control	treated	*P* value
RBC × 10^6^/μL	2.79 ± 0.026	1.87 ± 0.065	0.040
WBC(TLC) × 10^3^/μL	4.28 ± 0.008	6.92 ± 0.040	0.000
Hb (Hb) g/dL	11.87 ± 0.541	10.34 ± 0.362	0.078
HCT(PCV) %	29.27 ± 0.591	24.20 ± 1.105	0.016
Platelet count × 10^3^/ μL	3.39 ± 0.014	2.37 ± 0.366	0.049
MCH pg	38.33 ± 0.961	33.94 ± 0.718	0.022
MCHC pg	32.52 ± 1.171	27.79 ± 0.443	0.019
MCV fL	88.61 ± 0.531	80.18 ± 0.605	0.076

a All e values are expressed as mean ± S.E, based on five chickens per group. Statistical significance was determined using Student’s *t*-test: (*p* < 0.05) significant, (*p* < 0.01) more significant, and (*p* < 0.001) highly significant, compared to the control group. Abbreviations: RBC—Red blood cells (×10^6^/ μL), WBC (TLC)—White blood cells or total leukocyte count (×10^3^/ μL), Hb—Hb (g/dl), HCT (PCV)—Hematocrit or packed cell volume (%), PLT—Platelet count (×10^3^/ μL), MCH—Mean corpuscular Hb (pg), MCHC—Mean corpuscular Hb concentration (pg), MCV—Mean corpuscular volume (fL).

**11:** Hematological Blood Values in *G. gallus domesticus* after 40 Day Oral Exposure to 15 mg/kg Cd Compared to the Control Group^a^

parameters (units)	control	treated	*P* value
RBC × 10^6^/μL	3.11 ± 0.020	1.673 ± 0.0176	0.000
WBC(TLC) × 10^3^/μL	4.153 ± 0.022	7.24 ± 0.267	0.060
hemoglobin (Hb) g/dL	12.44 ± 0.291	10.06 ± 0.047	0.001
HCT(PCV) %	28.13 ± 1.180	21.23 ± 0.592	0.006
platelet count × 10^3^/μL	3.17 ± 0.115	2.13 ± 0.580	0.148
MCH pg	35.57 ± 0.550	31.24 ± 0.386	0.003
MCHC pg	33.52 ± 0.784	26.31 ± 0.373	0.001
MCV fL	86.22 ± 0.583	77.12 ± 0.539	0.043

a All values are expressed as mean ± S.E, based on five chickens per group. Statistical significance was determined using Student’s *t*-test: (*p* < 0.05) significant, (*p* < 0.01) more significant, and (*p* < 0.001) highly significant, compared to the control group. Abbreviations: RBC—Red blood cells (×10^6^/ μL), WBC (TLC)—White blood cells or total leukocyte count (×10^3^/ μL), Hb—Hb (g/dl), HCT (PCV)—Hematocrit or packed cell volume (%), PLT—Platelet count (×10^3^/μL), MCH—Mean corpuscular Hb (pg), MCHC—Mean corpuscular Hb concentration (pg), MCV—Mean corpuscular volume (fL).

**Table 12:** Hematological Blood Values in *G. gallus domesticus* after 60 Day Oral Exposure to 10 mg/kg Cd Compared to the Control Group^a^

parameters (units)	control	treated	*P* value
RBC × 10^6^/μL	3.03 ± 0.0305	1.79 ± 0.005	0.000
WBC(TLC) × 10^3^/μL	3.25 ± 0.560	6.16 ± 0.328	0.011
hemoglobin (Hb) g/dL	11.74 ± 0.257	6.373 ± 0.313	0.000
HCT(PCV) %	27.17 ± 0.583	21.93 ± 0.575	0.003
plateletcount × 10^3^/μL	3.56 ± 0.020	1.94 ± 0.032	0.000
MCH pg	37.33 ± 1.163	30.07 ± 0.566	0.005
MCHC pg	30.26 ± 0.452	23.63 ± 0.863	0.002
MCV fL	87.36 ± 0.645	74.62 ± 0.542	0.000

a All values are expressed as Mean ± S.E, based on five chickens per group. Statistical significance was determined using Student’s *t*-test: (*p* < 0.05) significant, (*p* < 0.01) more significant, and (*p* < 0.001) highly significant, compared to the control group. Abbreviations: RBC—Red blood cells (×10^6^/ μL), WBC (TLC)—White blood cells or total leukocyte count (×10^3^/μL), Hb—Hb (g/dL), HCT (PCV)—Hematocrit or packed cell volume (%), PLT—Platelet count (×10^3^/ μL), MCH—Mean corpuscular Hb (pg), MCHC—Mean corpuscular Hb concentration (pg), MCV—Mean corpuscular volume (fL).

#### 3.2.1 Effect on Red Blood Cell

In the acute exposure trials, the RBC count showed a nonsignificant increase after 3 days of Cd administration (2.88 ± 0.0519, *p* = 0.118) when compared to the control group. However, after 5 days of exposure, there was a significant increase in RBC count (3.74 ± 0.025, *p* < 0.05). This initial rise may reflect a compensatory hematopoietic response, possibly triggered by early oxidative stress, in which the body attempts to maintain adequate oxygen transport by increasing erythrocyte production or mobilizing stored erythrocytes from the spleen.

In contrast, a progressive and significant decrease in RBC count was observed during the chronic exposure phase. After 14 days, the RBC count dropped to 1.87 ± 0.065 (*p* < 0.05), followed by 2.45 ± 0.020 (*p* < 0.001) after 20 days, 1.673 ± 0.0176 (*p* < 0.01) after 40 days, and 1.79 ± 0.005 (*p* < 0.001) after 60 days of Cd exposure. This decrease strongly suggests the onset of Cd-induced anemia, which could be due to bone marrow suppression, impaired erythropoiesis, or increased destruction of circulating RBC. Cd is known to induce oxidative stress, which impairs the integrity of the RBC membrane and leads to hemolysis. It may also impair Hb synthesis by interfering with iron metabolism or inhibiting enzymes that are important for RBC maturation. The overall trend indicates that while acute exposure to Cd exposure may transiently stimulate erythropoiesis, prolonged exposure leads to significant hematological toxicity and anemia, compromising the oxygen-carrying capacity of the blood in Golden Misri chickens.

#### 3.2.2 Effect on White Blood Cell

The white blood cell (WBC) count showed a nonsignificant increase after 3 days of Cd exposure (4.98 ± 0.0115, *p* = 0.070), indicating the early onset of a mild immune response. This was followed by a statistically significant increase after 5 days (5.02 ± 0.008, *p* < 0.01), reflecting the onset of Cd-induced systemic stress and possible tissue damage that may have triggered immune activation.

With continued exposure, the WBC count remained elevated during the chronic trials. A significant rise was observed after 14 days (4.77 ± 0.115, *p* < 0.05) and became even more pronounced after 20 days (6.92 ± 0.040, *p* < 0.001), confirming sustained inflammatory or immunological stimulation. After 40 days, the WBC count remained elevated (7.24 ± 0.267, *p* = 0.060), although not statistically significant, suggesting a plateau effect or variability in the intensity of the immune response during this phase. After 60 days, there was again a significant increase (6.16 ± 0.328, *p* < 0.05), indicating a persistent leukocytosis in response to ongoing Cd toxicity.

These results suggest that Cd exposure stimulates an immune response, leading to increased WBC production likely as a defense mechanism against cell and tissue injury. Cd is known to promote the generation of reactive oxygen species (ROS), which leads to oxidative stress and inflammation that can activate leukocytes. The persistently high WBC levels observed over time could also be due to chronic inflammatory stress and dysregulation of the immune system. The nonsignificant increase after 40 days, despite previous sharp rises, could indicate an exhaustion of the immune system or a suppression of regulation during chronic toxicity. Overall, the data confirm that Cd triggers a dose- and time-dependent leukocytic response in Golden Misri chickens.

#### 3.2.3 Effect on Hemoglobin

In the acute exposure phase, Hb levels showed a significant increase after 3 days of Cd treatment (12.10 ± 0.208, *p* < 0.05), followed by a nonsignificant rise after 5 days (13.33 ± 0.318, *p* = 0.116) compared to the control group. This initial increase in Hb concentration could indicate a compensatory erythropoietic response, in which the body attempts to counteract oxidative stress or hypoxic effects induced by Cd by elevating Hb synthesis or releasing stored erythrocytes into the circulation. It could also reflect hem concentration due to reduced plasma volume under acute stress conditions.

In the chronic exposure trials, Hb levels showed a nonsignificant decline after 14 days (11.36 ± 0.279, *p* = 0.078) and after 20 days (10.34 ± 0.362, *p* = 0.078), suggesting the onset of Cd-induced suppression of erythropoiesis. The reductions became statistically significant after 40 days (10.12 ± 0.0176, *p* < 0.01) and were even more pronounced after 60 days (9.79 ± 0.005, *p* < 0.001), confirming the development of anemia associated with long-term Cd exposure.

The progressive decrease in Hb levels over time reflects the toxic interference of Cd with RBC synthesis and Hb production. Cd is known to interfere with iron metabolism and impair the function of key enzymes like δ-aminolevulinic acid dehydratase (ALAD) and ferrochelatase, both of which are important for hem biosynthesis. Moreover, chronic exposure to Cd leads to oxidative stress, which can damage the erythrocytes and shorten their lifespan, which in turn contributes to a decreasing Hb level. The marked drop observed after 60 days also suggests persistent bone marrow suppression or exhaustion of compensatory mechanisms and underlines the hematotoxic potential of prolonged Cd exposure in Golden Misri chickens.

#### 3.2.4 Effect on Hematocrit

Haematocrit values (HCT) showed a nonsignificant increase after 3 days (29.44 ± 0.558, *p* = 0.090) and 5 days (31.47 ± 0.669, *p* = 0.060) of Cd exposure compared to controls. This slight increase may be attributed to a transient hem concentration effect or transient stimulation of erythropoiesis, possibly reflecting the body’s early attempt to maintain adequate oxygen- transport capacity under acute toxic stress.

However, in the chronic exposure groups, HCT levels declined significantly, starting at 24.47 ± 0.263 (*p* < 0.01) after 14 days, followed by 24.20 ± 1.105 (*p* = 0.016) at 20 days, and dropping further to 21.23 ± 0.592 (*p* < 0.01) at 40 days, and 21.93 ± 0.575 (*p* < 0.01) at 60 days. This consistent downward trend strongly indicates the development of Cd-induced anemia.

The decline in HCT suggests a reduction in the proportion of RBC in whole blood, which may result from impaired bone marrow activity, suppressed erythropoiesis, or increased destruction of mature erythrocytes due to oxidative stress. Cd impairs hematopoietic stem cell differentiation and can also lead to damage to the erythrocyte membrane, resulting in hemolysis. The consistent reduction of HCT with increasing exposure duration highlights the cumulative hematotoxic effect of Cd in Golden Misri chickens.

#### 3.2.5 Effect on Platelet Count

The platelet count (PLT) showed a nonsignificant decrease after 3 days (3.56 ± 0.220, *p* = 0.676), 5 days (3.13 ± 0.227, *p* = 0.321), and 14 days (2.88 ± 0.075, *p* = 0.090), indicating that short-term exposure to Cd exposure does not cause immediate thrombocytopenia, although a downward trend was already noticeable.

However, after 20 days, however, a significant reduction in platelet count was recorded (2.37 ± 0.366, *p* < 0.01), revealing the onset of thrombocytopenia with prolonged Cd exposure. Although a nonsignificant decrease was observed after 40 days (2.13 ± 0.580, *p* = 0.148), the trend continued. A highly significant drop occurred after 60 days, with PLT levels falling to 1.94 ± 0.032 (*p* < 0.001), confirming the deleterious effects of chronic Cd toxicity on thrombopoiesis.

This progressive reduction in PLT may be attributed to bone marrow suppression, in which Cd interferes with the development of megakaryocytes—the precursor cells responsible for platelet formation. Additionally, the oxidative stress triggered by Cd can lead to increased destruction or elimination of PLT, which in turn contributes to thrombocytopenia. A low platelet count can compromise blood clotting ability and is another critical aspect of Cd-induced hematological dysfunction.

#### 3.2.6 Effect on Mean Corpuscular Hb

Mean corpuscular hemoglobin (MCH), which reflects the average amount of Hb per RBC, showed a nonsignificant increase during the acute phase of Cd exposure. After 3 days, the MCH value was 37.36 ± 1.132 (*p* = 0.427), and after 5 days 37.24 ± 0.574 (*p* = 0.082) compared to the control group. These values suggest that the initial exposure may transiently stimulate the Hb content per cell, possibly as part of a short-term compensatory response to oxidative stress or mild hypoxia.

However, during the chronic exposure period, MCH levels began to decline, reflecting progressive disruption of Hb synthesis. After 14 days, MCH dropped to 34.02 ± 0.560 (*p* = 0.186), although the change was not statistically significant. A significant reduction was noted after 20 days (33.94 ± 0.718, *p* < 0.05), and further declines were observed after 40 days (31.24 ± 0.386, *p* < 0.01) and after 60 days (30.07 ± 0.566, *p* < 0.01).

This declining trend in MCH demonstrates the toxic effect of Cd on Hb biosynthesis pathways, possibly through inhibition of key enzymes such as ALAD and ferrochelatase. The reduction in Hb per red cell, especially with prolonged exposure, indicates the development of hypochromic anemia, characterized by pale RBCs due to low Hb content. This confirms that Cd not only reduces the number of RBCs but also compromises their functionality.

#### 3.2.7 Effect on Mean Corpuscular Hemoglobin Concentration

Mean corpuscular hemoglobin concentration (MCHC), which measures the average concentration of Hb in a given volume of packed RBC, exhibited a nonsignificant increase during the acute exposure period. After 3 days, MCHC was 34.43 ± 1.132 (*p* = 0.323), and after 5 days it increased to 34.53 ± 0.946 (*p* = 0.181) compared to the control group. These slight increases could indicate early adaptive changes, possibly due to osmotic shifts or transient hemoncentration associated with acute Cd stress.

In the chronic exposure phase, however, a progressive and statistically significant decline in the MCHC was recorded. After 14 days, MCHC dropped to 28.17 ± 0.438 (*p* < 0.05), indicating the onset of hypochromic. The values fell further after 20 days to 27.79 ± 0.443 (*p* < 0.01), after 40 days to 26.31 ± 0.373 (*p* < 0.01), and after 60 days to 23.63 ± 0.863 (*p* < 0.01). These results clearly demonstrate a dose- and time-dependent reduction in the Hb concentration in the erythrocytes.

The steady decline in MCHC implies that Cd not only reduces the total Hb content (as shown in the Hb and MCH results), but also diminishes its packing density in RBC, a hallmark of hypochromic anemia. Mechanistically, this could be due to Cd’s inhibition of iron assimilation and Hb synthesis by Cd, as well as oxidative damage to the Hb molecules, which reduces their functional concentration in the cells. The severity of the MCHC drop after 60 days reflects a critical level of hematological impairment and illustrates the strong chronic hepatotoxicity of Cd.

#### 3.2.8 Effect on Mean Corpuscular Volume

Mean corpuscular volume (MCV), which reflects the average size of RBC, showed a nonsignificant increase in the acute phase of Cd exposure. After 3 days, MCV was 84.23 ± 1.132 (*p* = 0.296), and after 5 days, it slightly increased to 86.63 ± 1.148 (*p* = 0.193) compared to the control group. This early increase could indicate the presence of larger, immature erythrocytes (reticulocytes) in the bloodstream, possibly as a rapid response to oxidative stress or mild hypoxia caused by Cd exposure.

However, in the chronic exposure phase, MCV values began to decline. After 14 days, a significant reduction was observed (77.43 ± 0.617, *p* = 0.017), indicating the onset of microcytic changes in red the blood cells. The value after 20 days was 80.18 ± 0.605 (*p* = 0.076), showing a nonsignificant trend of continued reduction. As exposure progressed, the decrease again became statistically significant after 40 days (77.12 ± 0.539, *p* < 0.05), and even more pronounced after 60 days, with the MCV dropping to 74.62 ± 0.542 (*p* < 0.001).

This progressive reduction in MCV is consistent with the development of microcytic anemia, which is typically associated with disrupted Hb synthesis and iron deficiency—both of which may be the result of Cd toxicity. Cd has been reported to interfere with iron metabolism and the activity of enzymes necessary for red cell maturation. As a result, smaller RBCs with a lower Hb content are produced over time. The sharp drop in MCV after 60 days underscores the chronic suppression of erythropoiesis and the persistent damage to RBC integrity caused by Cd exposure.

## 4 Discussion

Exposure to Cd has profound impact on animal health, with Cd accumulating primarily in vital organs such as the liver and kidneys. In *G. gallus domesticus*, the most common domestic chicken species, exposure to varying concentrations of Cd chloride leads to maximum bioaccumulation in the liver and kidney. This accumulation is followed by the gizzard and intestine, while the deposition of Cd in muscle tissue remains minimal. These findings underscore the critical role of both dose and duration of exposure in determining the organ-specific Cd accumulation pattern. Our study is consistent with the observations of,^11^ that demonstrate the marked increase in Cd concentrations in the hepatic and renal tissue of chickens. This finding accentuates the liver’s pivotal role in detoxification processes, alongside the kidneys’ integral function in excretion, while further elucidating the limited metabolic activity of muscle tissue. In alignment with the investigations of^22^ and,^23^ we documented a considerable bioaccumulation of Cd in the liver and kidneys, while its presence in muscular tissue was negligible.^4^ identified the liver as the principal reservoir for Cd deposition, leading to corresponding concerns regarding the consumption of chicken liver. Similarly,^24^ described hepatic damage caused by Cd in hens exposed to elevated levels. Avian research has predominantly concentrated on the accumulation of Cd in the hepatic and renal systems, while little attention has been paid to other tissues.^11^ Our findings resonate with those of^25^ and,^26^ which corroborate the liver and kidneys as the primary sites of Cd uptake. Furthermore, ^11^also highlighted the concomitant bioaccumulation of lead and Cd in various chicken organs. In the present study, after peaking in the liver and kidneys, Cd accumulation was notably higher in the gizzard. This increased concentration may be attributed to the highly acidic environment of the gizzard (pH ∼ 2.0) and the uptake of gastroliths, which serve as a store for metals. This observation is consistent with the findings of,^27^ studies that similarly found elevated levels of Cd in the liver and gizzard of poultry. The intestines tends to accumulate lead and Cd, due to its efficient absorption mechanisms combined with suboptimal excretion capacities, as described by.^28^ Our findings, which are consistent with,^29^ emphasize that the gastrointestinal tract is a critical locus for heavy metal uptake. Conversely, muscle tissue has a lower affinity for heavy metals compared to hepatic and renal tissues, which limits metal uptake. Our results are also consistent with those of,^30^ confirming reduced lead and Cd concentrations in chicken muscle tissue. Haematological parameters serve as vital indicators of the general health status of *G. gallus domesticus*. Exposure to heavy metals, particularly Cd, leads to significant alterations in these indices. This study investigated the toxicological impact of Cd and showed that the extent of the effects depends on both the duration of exposure and the concentration. Acute exposure, specifically over 3 days (35 mg/kg) and 5 days (30 mg/kg), led to a significant increase in RBC, WBC, Hb, HCT, MCH, MCHC, and MCV values. In contrast, the platelet count decreased significantly compared to the control group, as presented in Table 2. Elevated RBC counts indicate hyperplasia of the bone marrow, while increased Hb concentrations reflect the compensatory response of the organism to improve oxygen uptake. The observed rise in MCV could be attributed to swelling of the erythrocytes, probably caused by osmotic stress or hypoxic conditions. Chronic exposure to heavy metals further exacerbates this condition by promoting the production of larger, immature erythrocytes (reticulocytes) with increased Hb content, thereby increasing both MCV and MCH.

Conversely, trials with prolonged exposure showed a remarkable reduction in RBC count, Hb, HCT, MCH, MCHC, MCV, and platelet count. However, the WBC counts were significantly increased compared to the control groups, as illustrated in Table 2. These findings are consistent with the research of^31^, which demonstrated that oral administration of CdCl_2_ in chickens leads to a significant decrease in HCT, Hb, MCH, MCHC, and MCV in all treated groups compared to controls. Similarly,^32^ documented a significant reduction in Hb, HCT, and erythrocyte count in mice exposed to Cd exposure.^33^ further documented these observations and showed that in chickens exposed to higher Cd concentrations (100 mg/kg) for 6 weeks, there was a significant (*P* ≤ 0.05) decrease in Hb, HCT, MCV, and RBC counts after 30 days of consumption of drinking water containing 10 mg/L Cd.^34^ documented that Cd acetate exposure in Kuroiler chicks resulted in significant alterations in hematological parameters, including reductions in RBC, Hb, HCT, MCHC, MCH, MCV, and platelet counts, with concomitant increases in WBC values. The observed declines in RBC, HCT, and Hb is indicative of anemia.^35^ proposed that the decrease in RBC count could be due to decreased erythropoiesis or hemorrhage induced by the toxin in the internal organs. Stress conditions further exacerbate the reduction in Hb content, RBC count, and HCT values, indicating a loss of red cells. Cd exposure induces oxidative stress and increases the activities of superoxide dismutase (SOD) and catalase in RBC*s*, leading to membrane damage, hemolysis, and hypoxia, as observed by^36^ in fish, and aligns with our findings. Additionally,^37^ and^38^ reported significant reductions in RBC, HCT, and Hb levels in rats exposed to high doses of Cd compared to controls.^19^ similarly noted decreases in MCH, MCHC, and MCV in chickens exposed to lead acetate and attributed these changes to diminished Hb concentrations, structural damage to RBC membranes, hemolysis, and disrupted Hb synthesis. The overall reduction in MCV, possibly due to the release of immature erythrocytes from the hematopoietic tissue in response to the loss of RBC, corroborates our findings. The decrease in platelet count observed in our study is consistent with the findings of ref 39, who reported a reduction in platelet levels in quail primarily attributed to bone marrow suppression induced by metals such as Cd. Cd exposure impairs thrombopoiesis by inhibiting platelet production in the bone marrow. Additionally, oxidative stress induced by Cd leads to damage and premature destruction of existing PLT. This stress also leads to the destruction of megakaryocytes, the precursor cells essential for platelet production, thereby further contributing to the reduction in platelet count. Our results are consistent with the observations of^40^, who found an increased WBC count in rats exposed to Cd chloride and attributed this increase to inflammatory responses. They also reported a decrease in Hb levels, probably due to the accumulation of metals in the RBCs and the inhibition of ferrochelatase, an enzyme critical for Hb synthesis. The increased number of WBC count observed in our study indicates a similar immune response to tissue damage, which may be a compensatory mechanism to mitigate the effects of Cd-induced stress and aid recovery. This substantial rise in WBCs supports our findings and highlights an immunological reaction to Cd exposure. It underscores the complex interplay between metal-induced oxidative stress, immune responses, and hematological changes and highlights the impact of Cd on blood health and overall physiological function.

## 5 Conclusion

This study provides important evidence of the hematological and bioaccumulative effects of cadmium (Cd) in Golden Misri chickens. The results confirm that Cd exposure leads to significant, dose- and duration-dependent accumulation in vital organs, particularly the liver and kidneys. While short-term exposure caused mild physiological changes, long-term exposure resulted in pronounced disruptions in hematological indices, including reductions in RBC, Hb, HCT, PLT, MCH, MCHC, and MCV—indicating the development of microcytic, hypochromic anemia and impaired hematopoiesis. In contrast, WBC levels increased significantly, suggesting a sustained inflammatory response and immune system activation. These findings highlight the sensitivity of hematological parameters as reliable early indicators of heavy metal toxicity. Given the widespread use of Golden Misri chickens in regional poultry farming and the risk of feed and water contamination, the study holds direct implications for poultry health, environmental toxicology, and food safety. The consistent relationship between Cd exposure, tissue accumulation, and hematological dysfunction underscores the need for continuous monitoring of heavy metals in poultry production systems. While this study was conducted under controlled conditions, further investigations across different poultry breeds and field settings are recommended to broaden the applicability of these findings and support informed risk assessment and regulatory efforts.

## Data Availability

All experimental supporting data and procedures are available within this article.

## Abbreviations

WBC: white blood cells
RBC: red blood cells
HGB: hemoglobin
HCT: hematocrit
PLT: platelets
MCH: mean corpuscular Hb
MCHC: mean corpuscular Hb concentration
MCV: mean corpuscular volume.
